# Positron emission tomography in ovarian cancer: 18F-deoxy-glucose and 16α-18F-fluoro-17β-estradiol PET

**DOI:** 10.1186/1757-2215-2-7

**Published:** 2009-06-16

**Authors:** Yoshio Yoshida, Tetsuji Kurokawa, Tetuya Tsujikawa, Hidehiko Okazawa, Fumikazu Kotsuji

**Affiliations:** 1 Department of Obstetrics and Gynecology, Faculty of Medical Sciences, University of Fukui, 23-3 Matsuoka-Shimoaizuki, Eiheiji-cho, Fukui, Japan; 2 Biomedical Imaging Research Center, Faculty of Medical Sciences, University of Fukui, 23-3 Matsuoka-Shimoaizuki, Eiheiji-cho, Fukui, Japan

## Abstract

The most frequently used molecular imaging technique is currently 18F-deoxy-glucose (FDG) positron emission tomography (PET). FDG-PET holds promise in the evaluation of recurrent or residual ovarian cancer when CA125 levels are rising and conventional imaging, such as ultrasound, CT, or MRI, is inconclusive or negative. Recently, integrated PET/CT, in which a full-ring-detector clinical PET scanner and a multidetector helical CT scanner are combined, has enabled the acquisition of both metabolic and anatomic imaging data using one device in a single diagnostic session. This can also provide precise anatomic localization of suspicious areas of increased FDG uptake and rule out false-positive PET findings. FDG-PET/CT is an accurate modality for assessing primary and recurrent ovarian cancer and may affect management. FDG-PET/CT may provide benefits for detection of recurrent of ovarian cancer and improve surgical planning. And FDG-PET has been shown to predict response to neoadjuvant chemotherapy and survival in advanced ovarian cancer. This review focuses on the role of FDG-PET and FDG-PET/CT in the management of patients with ovarian cancer. Recently, we have evaluated 16α-18F-fluoro-17β-estradiol (FES)-PET, which detects estrogen receptors. In a preliminary study we reported that FES-PET provides information useful for assessing ER status in advanced ovarian cancer. This new information may expand treatment choice for such patients.

## Background

Ovarian cancer is the second most common gynecologic malignancy. It has a relatively poor prognosis, accounting for approximately half of all deaths related to gynecologic cancer [1]. Conventional imaging with ultrasonography (US), computed tomography (CT) and magnetic resonance imaging (MRI) has been used, but ability to diagnose the primary tumor and accurately stage the ovarian cancer are variable. Such conventional imaging tools are also commonly used to guide the management of ovarian cancer patients. However, concerns remain that these imaging techniques may provide false negative results because of their inability to identify disease when normal anatomic landmarks have been lost because of surgery or radiation, or give false positive results related to their inability to distinguish between viable tumor masses and masses of necrotic or scar tissue [1-3]. New diagnostic imaging tools for primary and recurrent ovarian cancer have therefore been anticipated.

Functional imaging methods such as positron emission tomography (PET) can establish the metabolic or functional parameters of tissue. Instead of using anatomical deviations to identify areas of abnormality, PET uses positron-emitting radiolabeled molecules to display molecular interactions of biological processes *in vivo*. The most commonly used radioisotope tracer is 18F-deoxy-glucose (FDG), a glucose analog which is preferentially taken up by and retained within malignant cells. Depending on the area or organ under study, baseline glucose metabolism may be low, further highlighting the difference between normal background tissue and tumor [4]. However, FDG-PET has some limitations. It does not provide anatomic information, and precise localization of any suspicious lesions may accordingly be difficult. FDG-PET is also impaired by the presence of increased glucose uptake in physiologic, non-physiologic, or inflammatory states [4-9]. Recently, integrated PET/CT, in which a full-ring-detector clinical PET scanner and a multidetector helical CT scanner are combined, has enabled the acquisition of both metabolic and anatomic imaging data using one device in a single diagnostic session, and this provides precise anatomic localization of suspicious areas of increased FDG uptake and eliminates false-positive PET findings [9-15]. Bar-Shalom et al. demonstrated that FDG-PET/CT provided additional information compared with the separate interpretation of PET and CT in 178 of 53 sites (30%) imaged in 99 of 40 patients (49%). FDG-PET/CT improved characterization of equivocal lesions as definitely benign in 10% of sites and as definitely malignant in 5%. It precisely defined the anatomic location of malignant FDG uptake in 6% of sites, and it led to retrospective lesion detection on PET or CT in 8%. The results of FDG-PET/CT had an impact on the management of 28 patients (14%) whose management changed as a result of FDG-PET/CT, obviating the need for further evaluation in 5 (2%), guiding further diagnostic procedures in 7 (3%), and assisting in planning therapy for 16 patients (8%) [11]. Thus, compared with structural imaging techniques, FDG-PET and, moreover, FDG-PET/CT have the potential to deliver greater accuracy in diagnosis, staging, and management decisions in ovarian cancer.

In this review article, the role of FDG-PET and FDG-PET/CT in the diagnosis, staging, and management of ovarian cancer will be discussed. For conciseness we will focus on research published within the past decade and draw extensively on the texts and summaries of the articles referenced. Less recent citations are also included when deemed useful to provide background information.

16α-[^18^F]fluoro-17β-estradiol (FES) is a radiopharmaceutical that binds to the estrogen receptor (ER), thereby demonstrating the existence of this receptor [16]. FES can help diagnose ER-positive breast cancer and determine the efficacy of hormonal therapy in these patients [17]. In this article, we also discuss our preliminary studies indicating the usefulness of FES-PET imaging in the diagnosis of gynecologic cancer and in determining the efficacy of hormonal therapy [18,19], as a future PET method for evaluating ovarian cancer.

### Imaging protocol for ovarian tumors

FDG is excreted through the urinary tract and also physiologically accumulates in the bowel. Intense activity in the urinary or gastrointestinal tract can interfere with the optimal evaluation of the abdomen and pelvis. The most simple solution to this is to request that the patient empties their bladder just prior to imaging and to initiate imaging from the pelvis, before the bladder is full [8].

Other useful techniques to avoid false positives are bladder catheterization and furosemide administration. Koyama et al. reported that continuous bladder irrigation is useful for eliminating FDG activity in the bladder during FDG-PET (FDG activity in the urinary tract was eliminated in 80% of patients). The technique had satisfactory diagnostic utility with 100% sensitivity, 86% specificity and 98% accuracy for differentiating malignant from nonmalignant lesions. However, there is no foolproof method for avoiding bowel uptake [20].

It is now hoped that FDG-PET/CT will increase both sensitivity and specificity of PET by identifying physiologic tracer uptake and delineating cancerous lesions with low or absent FDG uptake [21]

#### Physiological FDG uptake in the ovaries

**Figure 1:**
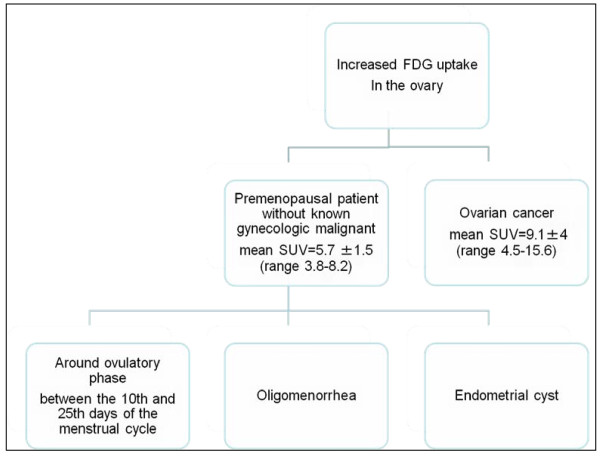
**A flow chart for differentiation of increased FDG uptake found incidentally**.

Increased physiologic ovarian FDG uptake in menstruating patients has been reported as an incidental finding. Lerman et al. evaluated patterns of FDG uptake during 4 phases of the menstrual cycle in 246 pre- and postmenopausal women without gynecologic tumors. Increased ovarian uptake was detected in 21 premenopausal patients, of whom 15 were at mid cycle and 3 reported oligomenorrhea. An ovarian standardized uptake value (SUV) of 7.9 differentiated benign from malignant uptake with a sensitivity of 57% and specificity of 95% [22]. Nishizawa et al. demonstrated focal ovarian FDG uptake in most premenopausal women examined 8 to 18 days before their next menstruation. This period corresponds roughly to the late follicular to early luteal phase. They also mentioned that physiological ovarian FDG uptake typically appeared as a round or oval area and was noted as an intense focal abnormality singular with a SUV greater than 3.0. It would therefore seem difficult to distinguish focal FDG uptake in the normal ovary from that in malignant lesions [23]. Moreover, Kim et al. demonstrated incidental ovarian FDG accumulations in 12 of 61 premenopausal women (20%), appearing between the 10th and 25th days of the menstrual cycle. No incidental FDG accumulations in the ovary were found in postmenopausal women. They concluded physiological ovarian FDG accumulation could be found around the time of ovulation and during the early luteal phase of the menstrual cycle in premenopausal woman [24]. The flow chart in Figure 1 summarizes the differentiation of increased FDG uptake found incidentally.

#### Screening for ovarian malignancy

Conventional morphological imaging modalities including US, CT, and MRI have been widely used to determine whether a suspicious ovarian tumor is malignant [1-3]. US performed in asymptomatic women as a screening test, followed by physical examination has a high sensitivity for differentiating malignant from benign ovarian processes, (82 – 96% in the literature, [25-27]), but specificity has varied widely among studies, from 52% to 93% [25-27].

Color and pulse Doppler techniques may aid in the diagnosis of ovarian cancer. Buy et al. compared gray-scale ultrasound with duplex and color Doppler in the evaluation of adnexal masses. Adding color Doppler to gray-scale morphologic information increased specificity from 82% to 97% and increased positive predictive value (PPV) from 63% to 97%, but duplex Doppler indices provided no further information [25-27]. A large meta-analysis comparing morphologic assessment, Doppler ultrasound, color Doppler flow imaging, and combined techniques for characterization of adnexal masses found combined techniques had the best diagnostic performance, followed in decreasing order by morphologic assessment alone, Doppler indices, and color Doppler [27].

CT and MRI have been utilized to further evaluate ovarian masses [1-3]. Although CT is more readily available and cost-effective than MRI, its usefulness in differentiating ovarian processes is limited because soft-tissue contrast is relatively poor when compared with MRI, and MRI therefore has higher diagnostic accuracy [1-3]. Reports in the literature differ with regard to the sensitivity and specificity of MRI in the differentiation of benign and malignant adnexal lesions, ranging from 85% to 95% for sensitivity and from 87% to 96% for specificity [28-31].

The sensitivity of FDG-PET in the detection of ovarian cancer was 78% in our study [32]; this was lower than the results reported in the literature, which have been in the range of 83% to 86% [33-37]. We suspect that the reason for the comparatively low sensitivity in our study was that our study population included a large number of false negative cases, such as patients with early mucinous adenocarcinoma and borderline mucinous adenocarcinoma. Rieber et al. reported that early carcinomas, mucinous adenocarcinomas, and particularly borderline tumors, present a problem because these tumors presumably lack the typical pattern of FDG uptake as a result of the small amount of transformed tissue [33], so they are likely to give false-negative results. Moreover, false-positive findings with FDG-PET occurred for endometriomas and dermoid cysts in our study. When the ovary is involved in an inflammatory process, inflammatory exudate may be accompanied by FDG uptake in these regions [34]. In addition, schwannomas, serous cystadenomas, thecomas, mucinous cystadenomas, and corpus luteum cysts show incidentally increased glucose metabolism has been reported in the literature [33-37].

In screening for ovarian cancer, US is the most important modality. MRI or FDG-PET, in addition to US, can provide further information about ovarian tumors and improve specificity. However, our study showed that the addition of FDG-PET to MRI does not yield additional information for the diagnosis of ovarian masses after US [32].

Recently, Castellucci et al. assessed the accuracy of FDG-PET/CT in distinguishing malignant from benign pelvic lesions, compared with transvaginal ultrasonography (TVUS). Adding FDG-PET/CT increased specificity from 61% to 100%, negative predictive value (NPV) from 78% to 81%, PPV from 80% to 100%, and accuracy from 80% to 92%. They concluded that FDG-PET/CT provides additional information to TVUS in the differential diagnosis of benign from malignant pelvic lesions [37]. In conclusion, US is the most important modality in screening for ovarian malignancy. Although some investigators consider FDG-PET useful in the differential diagnosis of malignancy, most studies have shown that it is of little value [32-36]. However, FDG-PET/CT may provide useful additional information when performed after TVUS in the differential diagnosis of malignancy [37].

#### PET in staging

A major problem in ovarian cancer is that a high proportion (75%) of patients have advanced stage disease at the time of diagnosis, which results in a 5-year survival rate of only 41% [1]. Primary debulking surgery is not the only treatment option for ovarian cancer, and patients with bulky, nonresectable disease will not benefit from primary surgery [1]. In addition, there is little survival benefit if the debulking is not optimal. The results of studies regarding therapy for patients with advanced cancer of the pancreas and esophagus provide clear evidence that neoadjuvant chemotherapy before surgery enables downstaging and thus improves operability as well as prognosis. The results of these studies strongly suggest the need to consider neoadjuvant chemotherapy in patients with advanced ovarian cancer [38]. Thereafter, accurate staging of patients with ovarian cancer before treatment is needed to determine appropriate treatment for those who will potentially benefit from it.

Our study is the first to show that the addition of FDG-PET to CT improves the staging accuracy of ovarian cancer [39]. The reason for this improvement was that FDG-PET facilitated detection of metastases outside the pelvis. For intrapelvic lesions, the sensitivity, specificity, PPV, NPV, and accuracy of CT alone increased from 72, 81, 48, 92, and 79%, respectively, to 76, 82, 50, 94, and 81%, respectively, when FDG-PET was added. Similarly, for lesions outside the pelvis, the sensitivity, specificity, PPV, NPV, and accuracy of CT alone increased from 24, 95, 44, 88, and 85%, respectively, to 63, 98, 88, 93, and 93%, respectively, with the addition of FDG-PET [39]. Although our study did not provide an evaluation on a per patient basis or a statistical analysis, to the best of our knowledge, it is the first to show that the addition of FDG-PET to CT improves the staging accuracy of ovarian cancer [39]. Recently, Kitajima et al. also reported that FDG-PET/contrast-enhanced CT was a more accurate imaging modality for staging ovarian cancer and was more useful for selecting appropriate treatment than enhanced CT alone [40].

In conclusion, FDG-PET is a useful and promising tool but not an established procedure in the staging of ovarian cancer patients. As studies in this field have been small-scale and have had variable results, a multicenter study with more data and showing clinical utility for routine use is needed before the procedure can be applied routinely for patients with confirmed or suspected ovarian cancer [33,39-43].

#### Diagnosis of recurrent ovarian cancer

Recurrent ovarian cancer is almost never curable, but early detection of recurrence theoretically increases the chance that salvage treatment will result in prolonged remission and sustained quality of life. Conventional imaging modalities often give nonspecific results and are suboptimal for the reliable detection of peritoneal recurrence. The identification of more accurate imaging modalities should improve management decisions for patients with recurrent ovarian cancer.

In 2002 we reported that FDG-PET was useful for following up an ovarian cancer patient in whom the only feature suspicious of recurrence was a rising CA125 level within the normal range [44]. Havrilesky et al. performed a meta-analysis to assess the diagnostic performance of FDG-PET in comparison with that of CT and MRI in patients with ovarian cancer. They concluded that FDG-PET did not appear to be useful in the routine surveillance of patients with a history of ovarian cancer, and that it was unlikely to improve the sensitivity of conventional modalities to detect microscopic intraperitoneal disease. There is fair evidence to support the use of PET for the detection of recurrent ovarian cancer when the CA-125 is elevated and conventional imaging is negative or equivocal, although whether this results in improved patient outcome is unclear [45].

The use of FDG-PET/CT for detecting recurrent ovarian cancer was first described by Makhija et al. in 2002 [46]. In 2008, Gu et al. performed a systemic meta-analysis to assess the accuracy of CA-125, PET alone, FDG-PET/CT, CT alone, and MRI in diagnosing recurrent ovarian carcinoma. They demonstrated that CA-125 had the highest pooled specificity, 0.93 (95%CI: 0.89 – 0.95), and FDG-PET/CT had the highest pooled sensitivity, 0.91 (95% CI: 0.88 – 0.94). They concluded FDG-PET/CT might be a useful supplement to current surveillance techniques, particularly for patients with an increasing CA-125 level and negative CT or MRI. However, regarding diagnostic accuracy, interpreted CT may have limited additional value over FDG-PET in detecting recurrent ovarian cancer [47]. Recently, Kitajima et al. reported that PET/contrast-enhanced CT was able to detect more malignant lesions than FDG-PET/CT or enhanced CT alone in recurrent ovarian cancer. Therefore, PET/contrast-enhanced CT could lead to changes in the subsequent clinical management of 39% of these patients. Improved diagnostic accuracy with PET/contrast-enhanced CT impacted management in 16 patients (12%) diagnosed by enhanced CT alone and in three patients (2%) diagnosed by PET/non-contrast-enhanced CT [48]. They concluded that PET/contrast-enhanced CT is an imaging modality with favorable accuracy for staging and for assessing ovarian cancer recurrence when compared with PET/non-contrast-enhanced or enhanced CT.

In conclusion, FDG-PET may provide benefits for those with elevated CA-125 (>35 U/ml), CT- or MRI-defined localized recurrence amenable to local destructive procedures, and clinically suspected recurrent or persistent cancer when biopsy cannot be performed. Using FDG-PET/CT or PET/contrast-enhanced CT is reported to have higher sensitivity and specificity than FDG-PET alone for detecting recurrent disease. We have summarized sensitivity and specificity for each imaging modality for the diagnosis of primary and recurrent/metastatic ovarian cancer in Tables 1 and 2.

**Table 1:** The following information shows the diagnosis of primary ovarian cancer

	Sensitivity	Specificity
Ultrasound (color and pulsed Doppler)	82%–96%	52%–93%
MRI	85% – 95%	87% – 96%
FDG-PET	58% – 86%	54% – 86%
PET-CT	88% – 100%	85% – 88%

**Table 2:** The following information shows the diagnosis of recurrent/metastatic ovarian cancer

	Sensitivity	Specificity
CT	40% – 91%	46% – 100%
MRI	55% – 91%	46% – 100%
FDG-PET	45% – 100%	50% – 100%
PET-CT	41% – 91%	71% – 100%

#### Usefulness of FDG-PET for assessing malignant activity

SUV is the most common PET parameter measured in the clinical setting. Its calculation is simple, and most contemporary FDG-PET/CT scanners display the imaging in these units, provided the injected dose and the patient weight have been entered when setting up the PET acquisition [49]. The role of SUV in PET examination has been discussed at length; however, doubts remain due to factors that can influence SUV calculation and reproducibility. A study by Nahmias et al. [49] investigated the reproducibility of SUV in malignant tumors and found that a number of factors other than the natural history of the tumor could cause variability in the measured SUV. These factors included fluctuations in plasma glucose and patient weight, errors in repositioning regions of interest (ROI) or image registration, and variations in the uptake period. They concluded that repeated measurements of mean SUV performed a few days apart were reproducible. A decrease of 0.5 SUV is statistically significant and may be considered when establishing thresholds to predict success of chemotherapy in patients with cancer.

We have previously assessed whether FDG-PET is useful for assessing malignant activity and gathering prognostic information in ovarian cancer [50]. We evaluated whether FDG uptake, quantified as SUV by PET in ovarian epithelial tumors, correlates with clinical stage [51,52], tumor grade [53], cell proliferation [54-56], or glucose metabolism [57], all of which are reported to be biomarkers for response to chemotherapy, prognosis, and overall survival in ovarian cancer patients. Epithelial ovarian tumor specimens were graded histopathologically, and immunohistochemistry for MIB-1 (a proliferation index marker) and GLUT-1 (glucose transporter marker) was performed. The correlations between FDG uptake and clinical stage, GLUT-1 expression, MIB-1 labeling index (LI), and histological grade were determined. No positive correlation was observed between FDG uptake and clinical stage (P = 0.14). On the other hand, the intensity of GLUT-1 expression (r = 0.76, P = 0.001), MIB-1 LI (r = 0.457, P = 0.014), and histological grade (r = 0.692, P = 0.005) showed statistically significant positive correlations with FDG uptake. Stepwise logistic regression analysis revealed that the expression of GLUT-1 transporters was the strongest predictor of positive FDG uptake (r = 0.760, P = 0.0004) [50].

A study of GLUT-1 expression in ovarian carcinoma by Canturia et al. showed that GLUT-1 status is an independent prognostic factor of response to chemotherapy in advanced ovarian carcinoma, and that patients over-expressing this marker have a significantly shorter disease-free survival rate [58]. Furthermore, Avril et al. showed that FDG-PET could predict response to neoadjuvant chemotherapy and survival in advanced ovarian cancer. Using a threshold for decrease in SUV from baseline of 20% after the first course, the median overall survival was found to be 38.3 months in responders (23.1 months in non-responders). At a threshold of 55% decrease in SUV after the third cycle, median overall survival was 38.9 months in responders (19.7 months in non-responders). Although the number of cases was small, this prospective study showed that sequential FDG-PET predicted patient outcome as early as after the first cycle of neoadjuvant chemotherapy and was more accurate than CA-125.[59]

In conclusion, glucose consumption, as determined by analysis of SUV in FDG-PET, may be a non-invasive biomarker that can predict response to chemotherapy and survival in ovarian cancer.

#### Cost-effectiveness evaluation of FDG-PET in the management of patients with ovarian cancer

Patients with advanced ovarian cancer who have completed a planned course of chemotherapy have frequently undergone a systematic surgical exploration and may be asymptomatic. About 36% to 73% of patients may have persistent disease detected at second-look procedures. Patients with residual disease should undergo continuous adjunctive therapy, while those without disease may discontinue adjunctive therapy. The cost-effectiveness and value of FDG-PET as a substitute for a second-look procedure have therefore been explored [14,60,61]. A detailed cost analysis of management of ovarian cancer with comparison of FDG-PET and second-look procedure was performed by Smith et al. [60]. They demonstrated that FDG-PET led to a decrease in the proportion of patients who underwent unnecessary laparotomy from 70% to 5%; 35% of patients underwent the less-invasive procedure of laparoscopy instead of laparatomy. Moreover, Kim et al. [61] reported the prognostic value of FDG-PET compared with that of a second-look procedure in patients with advanced ovarian cancer treated with chemotherapy. They concluded that PPV was 93% and NPV was 70% for FGD-PET, with no significant differences in progression-free interval between FDG-PET groups and second-look procedures. Hence FDG-PET appears to be useful and cost effective in the diagnosis of recurrent ovarian cancer.

#### A new PET tracer: potential applications in determining ER status

The sensitivity of ovarian cancer to hormonal therapy has a real, although modest, role in the treatment of advanced ovarian cancers resistant to chemotherapy. Many agents have been evaluated, including antiestrogens, estrogens, progesterones, androgens, aromatase inhibitors, and gonadotropin releasing hormone agonists (GnRH). As anticancer agents, hormonal therapies produce an approximate 10% response rate in previously treated patients. A correlation may exist between the presence of hormone receptors and a response to therapy [1]. Thus, knowledge of hormone receptor status, for example estrogen receptor (ER) status, is critically important for the treatment of ovarian cancer. Tissue sampling is essential but difficult because it is associated with significant morbidity and sampling error. The most commonly used molecular imaging technique in body imaging is currently FDG-PET. This has become the method of choice for staging and restaging in ovarian cancer, and it also has become extremely valuable in monitoring the response to anticancer agents. New PET agents, such as 16α-18F-fluoro-17β-estradiol (FES) have potential in the evaluation of response to hormonal therapy for ovarian cancer after FDG-PET. Although we do not have any experience of the use of FES-PET in patients on long-term hormone therapy to treat osteoporosis, we have already evaluated FES-PET for patients without any previous treatment in the differential diagnosis of benign and malignant uterine tumors [19].

**Figure 2:**
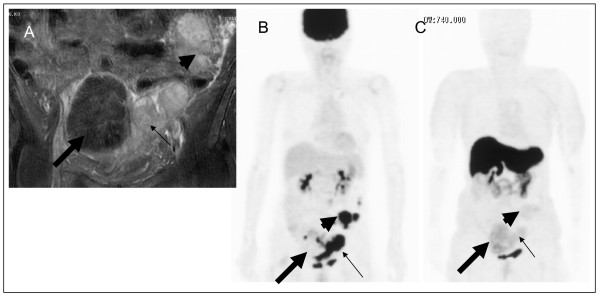
**A 66-year-old woman with a diagnosis of ovarian cancer and huge uterine leiomyoma underwent PET**. MRI demonstrated a huge uterine leiomyoma (large arrow) and left ovarian cancer (small arrow) with metastases in the abdomen (arrow head) (A). FDG-PET demonstrated ovarian cancer (small arrow) and multiple metastases in the abdomen and pelvis (arrow head), and a negative FDG-PET scan is shown for the leiomyoma (large arrow) (B). FES-PET demonstrated moderate uptake of FES in both the ovarian cancer (arrow head) and its metastases (arrow head) and leiomyoma (large arrow) (C).

**Figure 3:**
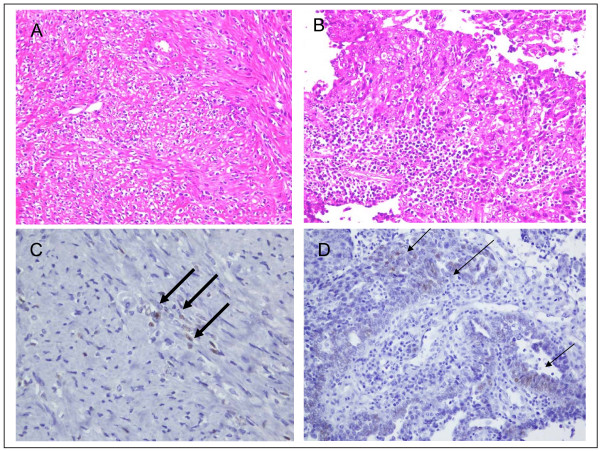
**Paraffin sections taken from the leiomyoma (A) and the ovarian cancer (B) demonstrate moderate ER-α expression**. The pattern of expression of ER-α in the leiomyoma (large arrow) (C) and ovarian cancer (small arrow) (D) was similar.

Here, we first showed that FES uptake was observed at primary and metastatic sites in three cases of advanced ovarian cancer. In these patients, we compared FES uptake and immunohistochemistry results for surgical specimens from patients with both primary and metastatic sites. These data indicated that FES uptake in PET was associated with ER status, particularly ER-α status, in ovarian cancer. A representative case was that of a 66-year-old woman with huge uterine leiomyoma and ovarian serous adenocarcinoma who underwent FES-PET before and after cytoreduction surgery. Before surgery, FES-PET showed moderately increased uptake in both the leiomyoma and ovarian cancer regions; the maximum SUV was 2.5 and 2.1 (figure 2), respectively. After resection, both the leiomyoma and ovarian cancer were found to be focally positive for estrogen receptor-α (ER-α) (figure 3). Hence FES uptake in PET was associated with ER-α status in ovarian cancer in this case. Although this study was only a preliminary case report, to the best of our knowledge it is the first to suggest that FES-PET could provide useful information about hormone status in advanced ovarian cancer. This information may be useful in expanding treatment choices for such patients.

## Conclusion

FDG-PET holds promise in the evaluation of cancer spread or recurrent or residual disease when other radiographic data are uncertain. FDG-PET/CT might be a useful supplemental investigation to detect primary and recurrent ovarian cancer earlier than FDG-PET and other conventional imaging tools. In addition, FES-PET may have the potential to provide useful information about hormone status in advanced ovarian cancer.

## Competing interests

The authors declare that they have no competing interests.

## Authors' contributions

YY drafted the manuscript. TK, TT, OH, and FK conceptualized, edited, and revised the manuscript. All authors have read and approved the final manuscript.
